# Geometrically focused training and evaluation of organs‐at‐risk segmentation via deep learning

**DOI:** 10.1002/mp.17840

**Published:** 2025-04-25

**Authors:** Ruiyan Ni, Elizabeth Chuk, Kathy Han, Jennifer Croke, Anthony Fyles, Jelena Lukovic, Michael Milosevic, Benjamin Haibe‐Kains, Alexandra Rink

**Affiliations:** ^1^ Department of Medical Biophysics University of Toronto Toronto Canada; ^2^ Princess Margaret Cancer Center University Health Network Toronto Canada; ^3^ Department of Radiation Oncology University of Toronto Toronto Canada; ^4^ Vector Institute for Artificial Intelligence Toronto Canada

**Keywords:** brachytherapy, cervical cancer, geometrically focused OAR delineation, image segmentation, new evaluation metric

## Abstract

**Background:**

Deep learning methods are promising in automating segmentation of organs at risk (OARs) in radiotherapy. However, the lack of a geometric indicator for dosimetry accuracy remains to be a problem. This issue is particularly pronounced in specific radiotherapy treatments where only the proximity of structures to the radiotherapy target affects the dose planning. In cervical cancer high dose‐rate (HDR) brachytherapy, treatment planning is motivated by limiting dose to the hottest 2 cubic centimeters (D2cm^3^) of the OARs. Similarly, Ethos online adaptive radiotherapy system prioritizes only the closest target structures for adaptive plan generation.

**Purpose:**

We propose a novel geometrically focused deep learning training method and evaluation metric, using cervical brachytherapy as a case study. A distance‐penalized (DP) loss function was developed to focus attention on the near‐to‐target OAR regions. We also introduced and evaluated a novel geometric metric, weighted dice similarity coefficient (wDSC), correlated with OARs D2cm^3^.

**Methods:**

A model was trained using a 3D U‐Net architecture and 170 T2‐weighted magnetic resonance (MR) images (56 patients) with clinical contours. The dataset was split into subsets at the patient level: 45 patients (150 scans) as the training set for five‐fold cross‐validation and 11 patients (20 scans) as the testing set. Another dataset from our institution, consisting of 35 MR scans from 22 cervical cancer patients, was used as an independent internal testing set. A distance map, emphasizing errors near high‐risk clinical target volume (CTV_HR_), was used to penalize two commonly used loss functions, cross‐entropy (CE) loss and DiceCE loss. The wDSC emphasizes the accuracy of OAR regions proximal to CTV_HR_ by incorporating a weighted factor in the original vDSC. The Pearson correlation coefficient (*r*) was used to quantify the strength of the relationship between D2cm^3^ accuracy and six evaluation metrics (wDSC and five standard metrics). A physician rated and revised the auto‐contours for the clinical acceptability tests.

**Results:**

The wDSC moderately correlated (*r* = ‐0.55) with D2cm^3^ accuracy, outperforming standard geometric metrics. Models using DP loss functions consistently yielded higher wDSCs compared to their respective non‐DP counterparts. DP loss models also improved D2cm^3^ accuracy, indicating an enhanced accuracy in dosimetry. The clinical acceptability tests revealed that more than 94% of bladder and rectum contours and approximately half of the sigmoid and small bowel contours were clinically accepted.

**Conclusion:**

We developed and evaluated a new geometric metric, wDSC, as a better indicator of D2cm^3^ accuracy, which has the potential to become a surrogate for dosimetric accuracy in cervical brachytherapy. The model with DP loss showed non‐statistically significant improvements in geometric and dosimetric performance. This work also holds the potential to be used for precise OARs delineation in adaptive radiotherapy.

## 1 INTRODUCTION

Contouring of organs‐at‐risk (OARs) and radiotherapy targets is a crucial step in radiation therapy, demanding intensive time of the radiation oncologists (ROs) and resources.^1^ With the emergence of computational and artificial intelligence technologies, automated contouring algorithms have been developed, including both atlas‐based^2^, ^3^ and deep learning‐based^4^, ^5^ approaches. However, the selection of robust evaluation methods for these algorithms is equally important. Given that accurate delineation of OARs and targets is fundamental to developing effective treatment plans,^6^ there is a clear need to emphasize meticulous assessment of these automated techniques prior to clinical deployment.

The performance of auto‐segmentation tools is commonly assessed using two geometric metrics.^7^, ^8^ The volumetric dice similarity coefficient (vDSC) evaluates accuracy by measuring the overlap between the auto‐segmented contours and the ground truth. The 95% Hausdorff Distance (HD95) quantifies discrepancies at the segmentation boundaries. While these metrics have been widely utilized and are considered the “gold standard” for model comparisons, they have been found to have certain pitfalls^9^ and do not necessarily align with clinical acceptance.^10^

Novel evaluation metrics have been proposed in previous studies for better clinical contour quality assessment.^11^, ^12^ Surface DSC (sDSC), derived from the vDSC, quantifies the OARs surface contours instead of volumes to better reflect the manual corrections.^12^ Added path length (APL) measures the path length that needs to be added to match the ground truth contours.^11^ Both sDSC and APL have been demonstrated to be more correlated with clinical time‐saving than traditional metrics.^11^, ^13^ Yet, a comprehensive investigation of the relationship between geometric measurements and dosimetric accuracy remains to be conducted as the ultimate goal of contour delineation is to guide the treatment plans for improved tumor control and reduced toxicity.

In certain forms of radiotherapy, the relationship between the existing geometric evaluation metrics and the clinical relevance is further diminished, as the distance between the OARs and the targets needs to be carefully accounted for. In adaptive radiotherapy, organs in closest proximity to the target require precise segmentation, as they have the greatest influence on daily clinical target structures and, consequently, on dosimetry.^14^ Similarly, in brachytherapy, since OAR dose constraints for brachytherapy are driven by the minimum dose in the most irradiated 2 cm^3^ of tissue volume (D2cm^3^) adjacent to the applicator.^15^ This means that the contouring accuracy must be high for the OAR regions proximate to the target, while discrepancies in distant regions may not translate to significant dosimetric or clinical impacts. In this study, we used cervical brachytherapy as an example to demonstrate a new metric and a deep learning training method tailored for auto‐segmentation in near‐to‐target OARs.

This study proposes a new evaluation metric, weighted DSC (wDSC), by weighting the vDSC with voxel distance to the high‐risk clinical target volume (CTV_HR_). We assessed the performance of five standard geometric metrics alongside wDSC in terms of their correlation with dosimetric accuracy where our newly proposed metric, wDSC, demonstrated the strongest correlation. Additionally, we introduce a distance‐penalized (DP) loss function^16^, ^17^ into a deep learning model training to enhance the segmentation accuracy of near‐to‐target OAR segmentation. We thoroughly assessed the quality of the automatic segmentations through geometric evaluations, dosimetric analysis between automatic and expert delineations, and clinical acceptability tests. The methods and metrics described can be applied to prostate brachytherapy as well as online adaptive radiotherapy.

## 2 METHODS

**FIGURE 1:**
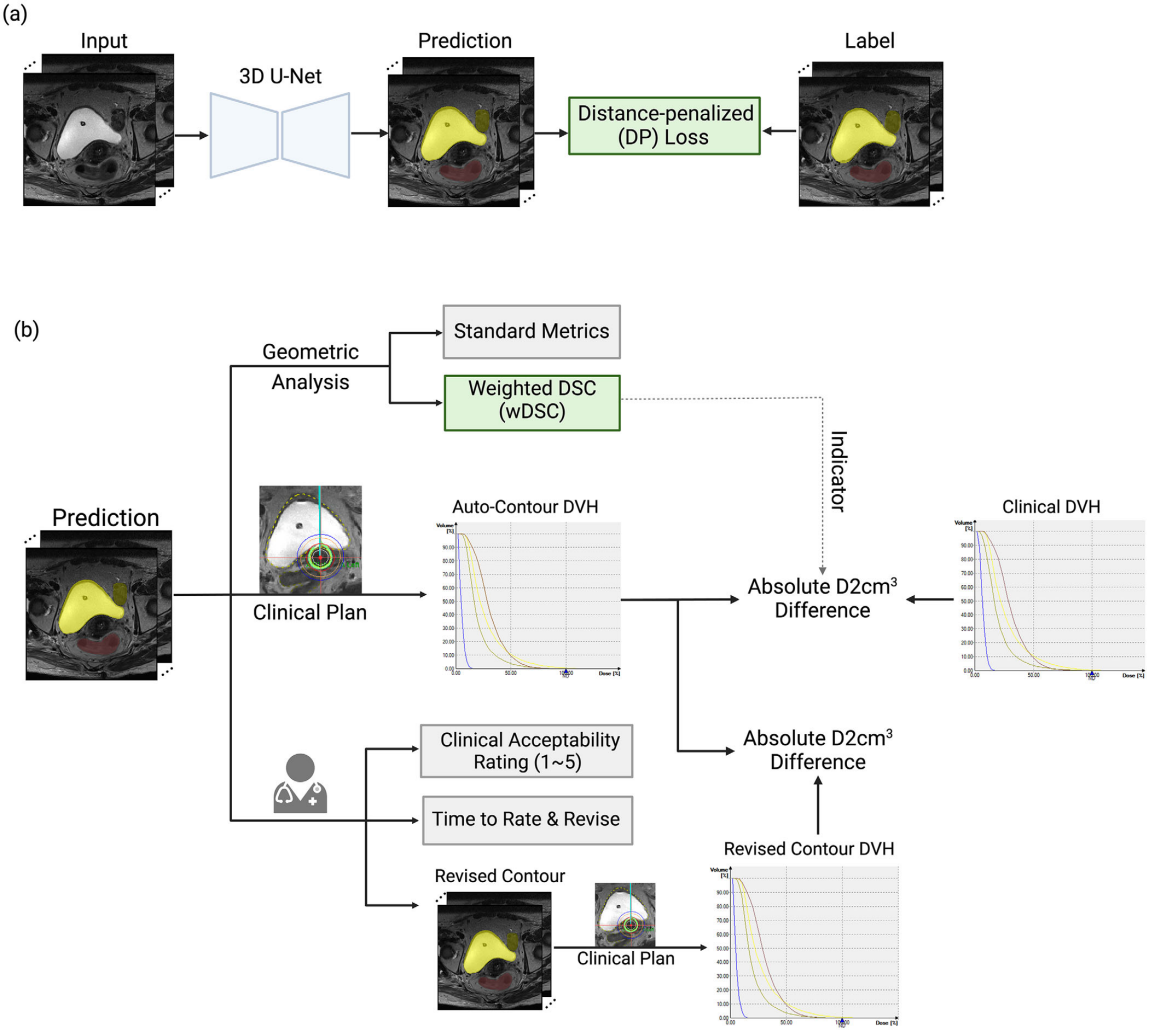
Schematic of study design. (a) Network training with DP loss. (b) Evaluation of predictions through geometric analysis (top), dosimetric evaluation (middle), and clinical acceptability test (bottom).

The overall study design is outlined in Figure 1. We employed a 3D U‐Net architecture, training the model using both the proposed distance penalized (DP) loss function and a non‐DP loss function for comparative analysis. The evaluation included geometric analysis, dosimetric evaluation, and clinical acceptability testing.

### 2.1 Patient data

A dataset of 170 T2‐weighted magnetic resonance (MR) scans with clinically used contours was built from 56 patients undergoing external beam radiotherapy (EBRT) and high‐dose‐rate (HDR) brachytherapy between 2014 and 2019 at our institution with Institutional Review Board (IRB) approval. Patient demographics, including year treated, T category, N category, FIGO 2018 stage, pelvic EBRT dose, and prescribed brachytherapy dose, are summarized in Table 1. Each patient received anesthesia prior to insertion of a bladder Foley catheter and ring and tandem applicators (Elekta, Stockholm, Sweden), with or without interstitial needles. Patients were imaged after applicator insertion using a Verio 3T MR scanner (Siemens, Munich, Germany). Depending on the procedure, the scanning was conducted while the patients were either awake or anesthetized. The median image size was 320 × 320 × 38 voxels, and the median image spacing was 0.625 × 0.625 × 3.0 mm^3^. Clinical contours used in this study, reviewed by experienced ROs, included the CTV_HR_, bladder, rectum, sigmoid, and small bowel. All the OARs were contoured as whole organs in accordance with the ICRU89^18^ guideline. The dataset was split into subsets at the patient level: 45 patients (150 scans) as the training set for five‐fold cross‐validation and 11 patients (20 scans) as the held‐out testing set.

**TABLE 1:** Summary of datasets. One hundred seventy scans from 56 patients were used in the primary dataset for training, validation, and held‐out testing; 35 scans from 22 patients were used as independent internal testing. Individual patients might have had multiple scans.

	Primary dataset	Independent internal testing dataset
Year	2014–2019	2017–2021
FIGO Stage (# of patients)		
IB	9	/
II	24	4
III	21	13
IVA	2	5
T Category (# of patients)		
T1	11	/
T2	39	8
T3	4	9
T4	2	5
Pelvic lymphadenopathy (# of patients)	21	12
Paraaortic adenopathy (# of patients)	5	4
Pelvic EBRT dose (Gy)	45 Gy/25 fx or 50.4Gy/28fx	45 Gy/25 fx
Brachytherapy dose (Gy)	7 Gy × 4fx or 8 Gy × 3fx	7 Gy × 4fx or 8 Gy × 3fx
Applicator used	Ring & Tandem ± needles	Syed‐Neblett template
Scanner used	3T	1.5 T

Another dataset from our institution, consisting of 35 MR scans from 22 cervical cancer patients, was used as an independent internal testing set^19^ to further evaluate the generalizability of the model with the proposed DP loss function. Patient information is summarized in Table 1. Patients in this dataset were treated using the Syed‐Neblett template (Best Medical, Virginia, USA) and scanned with an Espree 1.5 MR scanner (Siemens, Munich, Germany). Of the 35 scans, 25 were used for model fine‐tuning and the remaining 10 scans were used for independent internal testing.

### 2.2 Distance‐penalized loss function

**FIGURE 2:**
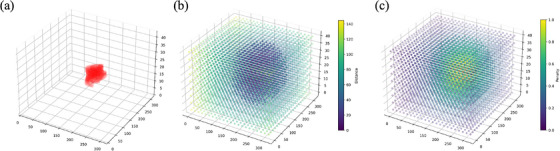
Examples of (a) CTV_HR_, (b) Distance transformation based on CTV_HR_, and (c) Inverse square law map (distance‐penalized map). Points in (b) and (c) are downsampled with slice intervals of (15, 15, 5) along (x, y, z).

Distance‐penalized maps, indicating spatial relevance through labeling which voxels were near the target/applicator, were generated to penalize the loss function. Distance transformation^20^ was performed on a binary mask, with the clinically used CTV_HR_ (Figure 2a) labeled as 1, calculating the distance between each voxel to its nearest non‐zero voxel, as illustrated in Figure 2b. Photon fluence from a brachytherapy source, and thus dose, can be modeled through inverse square law to its first approximation as shown in Equation (1),

(1) $$P=\frac{1}{R^{2}}\;$$

where R is the distance (in mm) from CTV_HR_ to one voxel. The DP maps (Figure 2c) were generated by normalizing P to lie within the range [0, 1]. Consequently, voxels in closer proximity to the CTV_HR_ would be assigned higher error penalties.

The DP map was used to penalize two commonly used loss functions, cross‐entropy (CE) loss and Dice loss, which are defined by

(2) $$L_{CE}=-\frac{1}{N}\sum_{c=1}^{C}\sum_{i=1}^{N}g_{i}^{c}logp_{i}^{c}$$

(3) $$L_{Dice}=1-\frac{2\sum_{c=1}^{C}\sum_{i=1}^{N}g_{i}^{c}p_{i}^{c}}{\sum_{c=1}^{C}\sum_{i=1}^{N}g_{i}^{c}+\sum_{c=1}^{C}\sum_{i=1}^{N}p_{i}^{c}}$$

where $g_{i}^{c}$ and $p_{i}^{c}$ are the ground truth label and the network prediction of class 𝑐 at voxel 𝑖. 𝑁 and 𝐶 are the number of voxels and classes, respectively. A “class” refers to a label that each voxel in a volume can belong to, such as background, or one of the four OARs. To smooth training, we used DiceCE loss, a combo loss function that integrates Dice and CE loss in our experiment.^17^, ^21^, ^22^ It is defined as follows:

(4) $$L_{DiceCE}=L_{Dice}+L_{CE}$$

The penalized loss functions, distance‐penalized cross‐entropy (DPCE) loss and distance‐penalized Dice (DPDice) loss, are defined by

(5) $$L_{DPCE}=-\frac{1}{N}\sum_{c=1}^{C}D^{c}⊙\;\sum_{i=1}^{N}g_{i}^{c}logp_{i}^{c}$$

(6) $$L_{DPDiceCE}=1-\frac{2\sum_{c=1}^{C}D^{c}⊙\sum_{i=1}^{N}g_{i}^{c}p_{i}^{c}}{\sum_{c=1}^{C}\sum_{i=1}^{N}g_{i}^{c}+\sum_{c=1}^{C}\sum_{i=1}^{N}p_{i}^{c}}+L_{CE\;}$$

where $D^{c}$ is the distance penalty term of class c from DP map. ⊙ is the Hadamard product. By incorporating the DP map into the loss function, the pixels that are closer to CTV_HR_ are assigned larger weights.

### 2.3 Network architecture and training procedure

The constructed network pipeline (grounded in nnU‐Net^21^) employed a 3D full‐resolution U‐Net model. Four models were trained using two baseline loss functions (CE loss and Dice loss) and two DP loss functions (DPCE loss and DPDiceCE loss). Optimization was achieved through a stochastic gradient descent (SGD) optimizer, accompanied by an initial learning rate of 0.01 and Nesterov momentum parameterized at 0.99. Data augmentation strategies used in the training set encompassed scaling (factor = 0.7‐1.4), Gaussian noise and Gaussian blur insertion (probability = 0.3), and mirroring. The patch size was 256 × 256 × 28, and the batch size was 2. The models were trained for 200 epochs to balance learning efficacy and mitigate the risk of overfitting.

For the independent internal testing, our previous study demonstrated that a model trained on data from a specific applicator and scanner cannot be directly applied to data acquired with different applicators and scanners.^19^ Therefore, the primary model was first fine‐tuned for a minimum of 20 epochs until convergence. Early stopping was applied, terminating training if the validation loss showed no improvement for five consecutive epochs after the initial 20 epochs. The fine‐tuned models were then evaluated on the 10 testing scans in the independent internal set.

The proposed framework was implemented in Python 3.9.7 using PyTorch 1.11.0. The training and testing were conducted using an NVIDIA Tesla V100 graphics processing unit (GPU) with 32GB memory. The training and testing code is available on GitHub: https://github.com/bhklab/BrachyNewLoss.

### 2.4 Geometric evaluation

#### 2.4.1 Standard metrics

The auto‐segmentations were compared to the manual segmentations (ground truth) using five evaluation metrics: vDSC, HD95, sDSC,^15^, ^18^ APL,^22^, ^23^ and averaged symmetric surface distance (ASSD).^23^ The vDSC is defined as

(7) $$vDSC=\frac{2\left(V_{G}\;\cap \;V_{p}\right)}{V_{G}+V_{P}}\;$$

where $V_{G}$ is the volume of ground truth segmentation, and $V_{P}$ is the volume of network prediction. HD95 computes the maximum distance among the 95th percentile of the nearest‐neighbor point distances from one set to the other. ASSD is calculated as the average of all the shortest distances from each point on the surface of one contour to the surface of the other contour, measured bidirectionally.

sDSC calculates the overlap between the surfaces of the manual contour and network output, utilizing the same equation as vDSC (Equation 7) with a tolerance of 1 mm. APL measures the number of pixels present on the surface of the ground truth contour but absent from the prediction and is defined by

(8) $$APL=S_{G}\;-\left(S_{G}\cap S_{P}\right)$$

where $S_{G}$ and $S_{P}$ are the surfaces of ground truth and prediction, respectively. The metrics were implemented using a Python package (https://github.com/deepmind/surface‐distance).

#### 2.4.2 New metric‐weighted DSC

**FIGURE 3:**
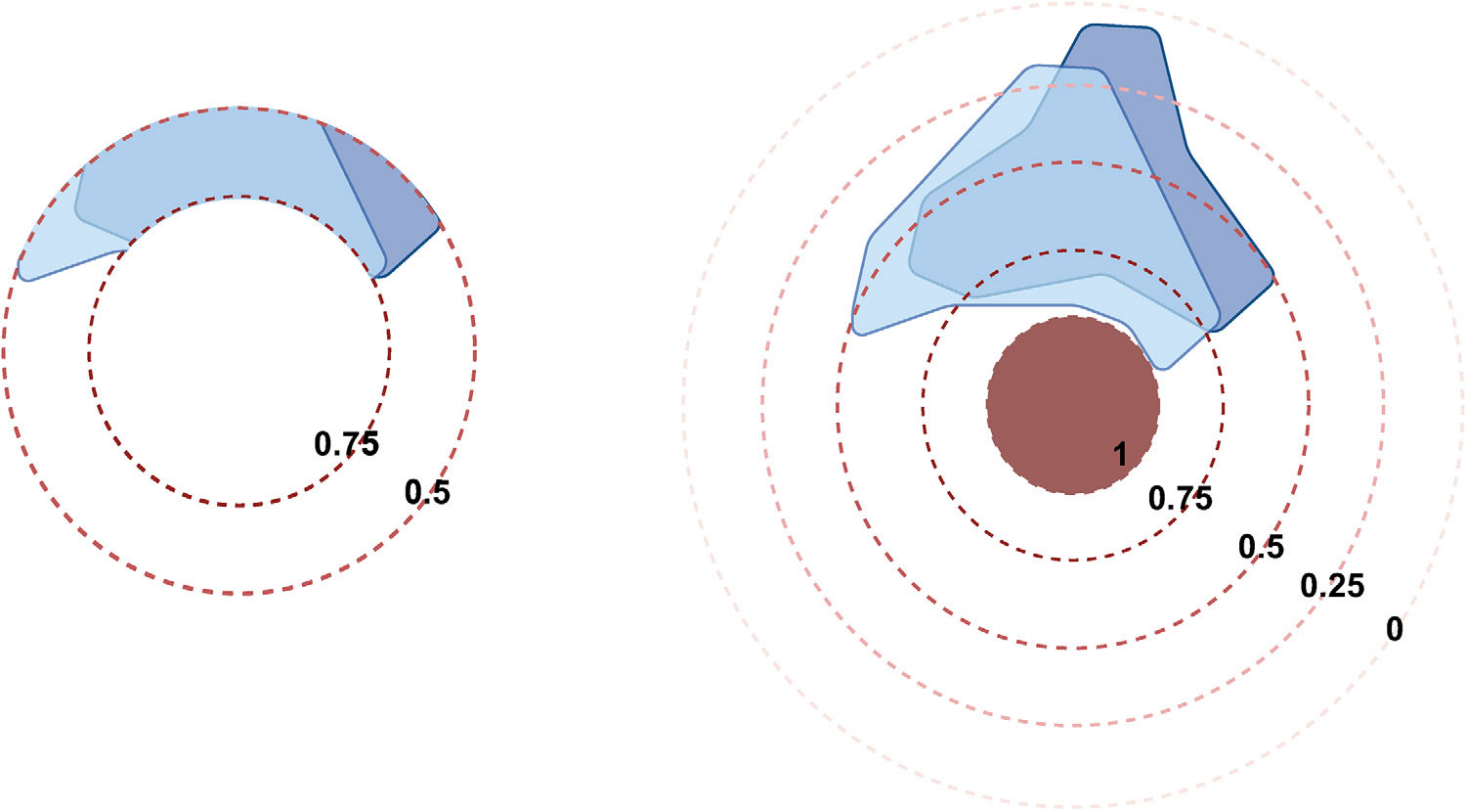
The illustration of wDSC between ground truth (lighter blue) and network prediction (darker blue). (a) Both are first cropped into N volumes based on the values in the distance map. In this example, N= 4; the weights of each cropped volume = (1, 0.75, 0.5, 0.25). (b) A zoomed‐in version of volumes with weight = 0.75. Assuming vDSC=0.8 between these two cropped volumes, the weighted vDSC loss is 0.75*(1‐0.8). The final wDSC is $1\;-\;\frac{total\;weighted\;vDSC\;loss}{N}$, iterating through all cropped volumes. From the boundary to the center, the weights are $\left(\frac{1}{N},\frac{2}{N},\text{…},\frac{N-1}{N},1\right)$. In this study, we used N= 100.

We introduced a new evaluation metric, weighted DSC (wDSC), specifically designed as an indicator for near‐to‐target OAR segmentation performance. Given that D2cm^3^ measures the minimum dose to the most irradiated 2cm^3^ volumes of OARs nearest to the radiotherapy target, the wDSC emphasizes the accuracy of proximal OAR regions to CTV_HR_ by incorporating a weighted factor in the original vDSC. The weights are derived from the voxel distances to CTV_HR_, as represented in the DP map. The wDSC is defined by

(9) $$wDSC=1-\;\frac{\sum_{i=1}^{N}w_{i}*\left(1-vDSC_{i}\right)}{N}$$

where N is the total number of cropped volumes; $vDSC_{i}$ is the vDSC between the i‐th cropped volumes of label and prediction; $w_{i}$ is the weight of the i‐th cropped volume, calculated as $w_{i}$ = $\frac{i}{N}$. As shown in the illustration (Figure 3), cropping occurs based on the DP map, starting from the outer boundary and progressing toward the center. Each cropped volume is assigned a weight that increases as it moves closer to the center. Consequently, discrepancies between the prediction and ground truth near the target center have a greater influence on the wDSC value than those farther away. For each cropped volume, the weighted discrepancy is calculated as $w_{i}*\left(1-vDSC_{i}\right)$, which is then summed across all the cropped volumes. The summed discrepancy is normalized by dividing by N (the total number of cropped volumes). The final wDSC is obtained by subtracting the normalized discrepancy from 1. In this study, we used N= 100 to crop each scan into 100 cropped volumes, where each cropped volume was small enough for the weighting to be considered continuous. A Wilcoxon signed‐rank test was utilized to analyze the statistical differences in the wDSCs between models trained with CE and DPCE, as well as between models trained with DiceCE and DPDiceCE loss functions, to determine the effectiveness of incorporating a distance penalty term in improving the delineation of near‐to‐target OARs. We utilized the Bonferroni correction to modify the results of our statistical tests, setting the adjusted significance threshold at 0.00625.

### 2.5 Dosimetric evaluation

The dosimetric differences between manual contours and DL‐generated contours were calculated for each fraction. The auto‐segmentations were imported into Oncentra Brachy v4.6 (Elekta, Stockholm, Sweden) in DICOM RTStruct format. The clinical applicator/needle reconstruction and treatment plan were applied to the auto‐segmentations. The cumulative dose‐volume histogram (DVH) was generated for each contour, and D2cm^3^ for OARs was extracted. The DVH parameters were set as 100,000 sample points, 200 pins, and high dose limits of 4. The OAR dose constraint, D2cm^3^, was calculated as the minimum dose in the most irradiated 2 cm^3^ of tissue volume. The absolute D2cm^3^ difference was quantified as

(10) $$D2cm_{\mathrm{diff}}^{3}=\;\mathrm{abs}\left(D2cm_{\mathrm{DL}}^{3}-D2cm_{\mathrm{clin}\mathrm{ical}}^{3}\right)$$

indicating the discrepancy between auto‐contour ($D2cm_{DL}^{3}$) and clinical contour D2cm^3^ ($D2cm_{clinical}^{3}$). An absolute $D2cm_{diff}^{3}$ value closer to zero indicated that the auto‐contour aligned better with the dosimetric requirements, suggesting that the automated contouring is sufficiently accurate compared to the clinical contouring.

The correlations between each geometric evaluation metric and the absolute D2cm^3^ difference (all values combined across the four trained models) were evaluated to explore the best indicator of dosimetric accuracy. Linear regression was performed, employing the Pearson correlation coefficients to quantify the strength of the relationship. Wilcoxon signed‐rank test with the Bonferroni‐corrected *p*‐value of 0.00625 was used to analyze the statistical differences between the clinical D2cm^3^ values and the D2cm^3^ values obtained from each loss function.

### 2.6 Clinical acceptability test

A gynecologic radiation oncology fellow rated the DL contours on a five‐point scale^24^ (Table S1). The fellow revised the auto‐contours if it initially failed to meet clinical standards. The time taken for rating and revising was recorded. The dose parameters were derived from revised contours to assess the impact of expert revisions on treatment efficacy.

## 3 RESULTS

### 3.1 Correlations between evaluation metrics and D2cm^3^ difference

**FIGURE 4:**
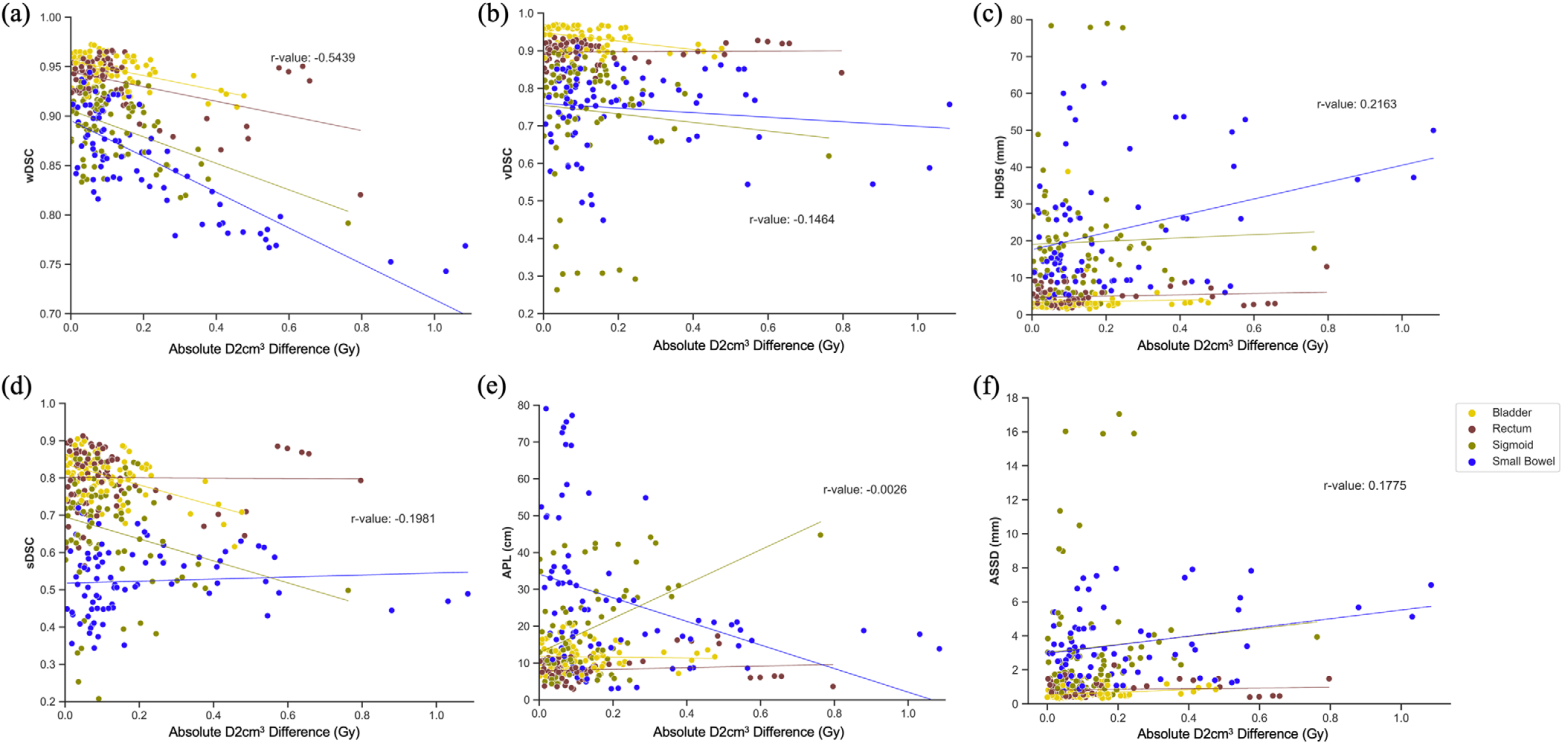
Correlations between absolute D2cm^3^ difference and (a) wDSC, (b) vDSC, (c) sDSC, (d) HD95 (mm), (e) APL (cm), and (f) ASSD (mm). Bladder (yellow), rectum (brown), sigmoid (yellow green), and small bowel (blue) are combined over all four models. ‘r‐value’ is the Pearson correlation coefficient. wDSC and D2cm^3^ are moderately correlated. wDSC: weight Dice Similarity Coefficient; vDSC: volumetric DSC; sDSC: surface DSC; HD95: 95% hausdorff distance; APL: added path length; ASSD: average symmetric surface distance.

We performed the Pearson correlation analysis to investigate the correlations between each evaluation metric and the D2cm^3^ difference. The strongest correlation between absolute D2cm^3^ difference and all geometric evaluation metrics was for the novel metric, wDSC, with a Pearson correlation coefficient (*r*) of −0.54, indicating a moderate negative correlation. In contrast, the other five standard geometric evaluation metrics demonstrated weak or negligible correlations with the D2cm^3^ difference (*r* of −0.15 for vDSC, 0.22 for HD95, −0.20 for sDSC, −0.003 for APL, and 0.18 for ASSD) (Figure 4).

### 3.2 Geometric evaluation

**FIGURE 5:**
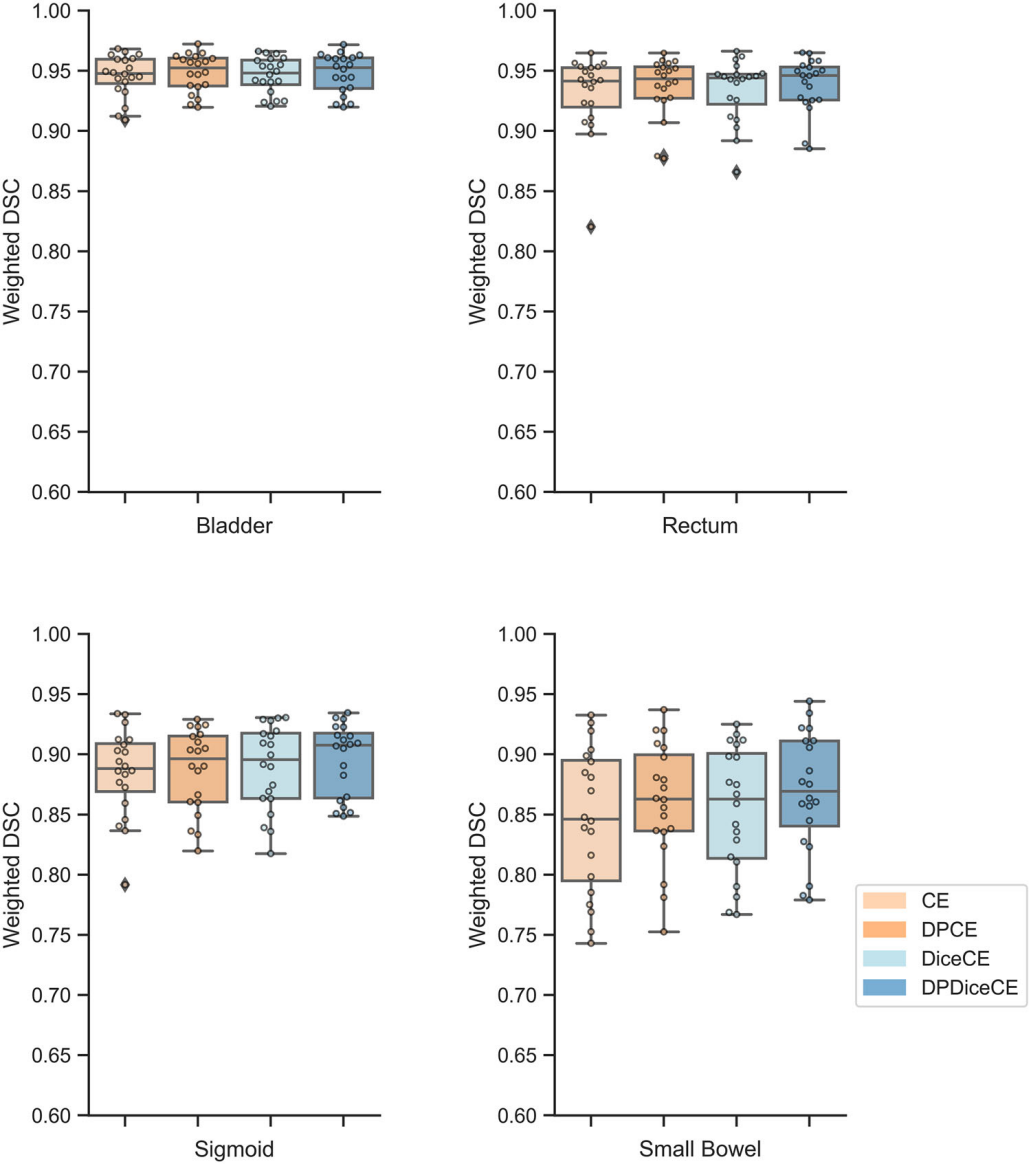
Weighted DSCs (wDSCs) comparison of CE and DPCE (orange), as well as of DiceCE and DPDiceCE (blue). In each box and whisker plot, the box represents the interquartile range (IQR); the upper and lower whiskers show values outside the middle 50%.

The newly proposed metric, wDSC, indicated that the model trained with the DP loss functions consistently yielded more accurate near‐to‐target OAR segmentation for small bowel when compared to their respective non‐DP counterparts (Figure 5). DPCE loss had small non‐significant improvements compared to CE loss for rectum (mean wDSCs: 0.94 and 0.93), sigmoid (mean wDSCs: 0.89 and 0.88), and small bowel (mean wDSCs: 0.86 and 0.85). Similarly, DPDiceCE had increased wDSCs, compared to DiceCE, for rectum (mean wDSCs of DPDiceCE and DiceCE: 0.94 and 0.93), sigmoid (0.90 and 0.89), and small bowel (0.87 and 0.85). Mean wDSCs of the bladder remained the same for all four loss function groups (mean wDSC = 0.95). The model trained with the DP loss functions demonstrated non‐significant improvement in the small bowel compared to the ones with the original loss functions (mean wDSC: 0.86 and 0.84 with *p *= 0.008 in the CE group; mean wDSC: 0.87 and 0.85 with *p *= 0.017 in the DiceCE group).

The DP group outperformed the non‐DP group across five standard metrics (Table S2). The most notable improvement was observed between DiceCE and DPDiceCE in small bowel with mean vDSCs of 0.74 and 0.77, mean HD95 of 23.95 and 19.04 mm, mean sDSCs of 0.52 and 0.55, mean APL of 27.69 and 27.01 cm, and mean ASSD of 3.67 and 2.98 mm.

### 3.3 Dosimetric evaluation

**FIGURE 6:**
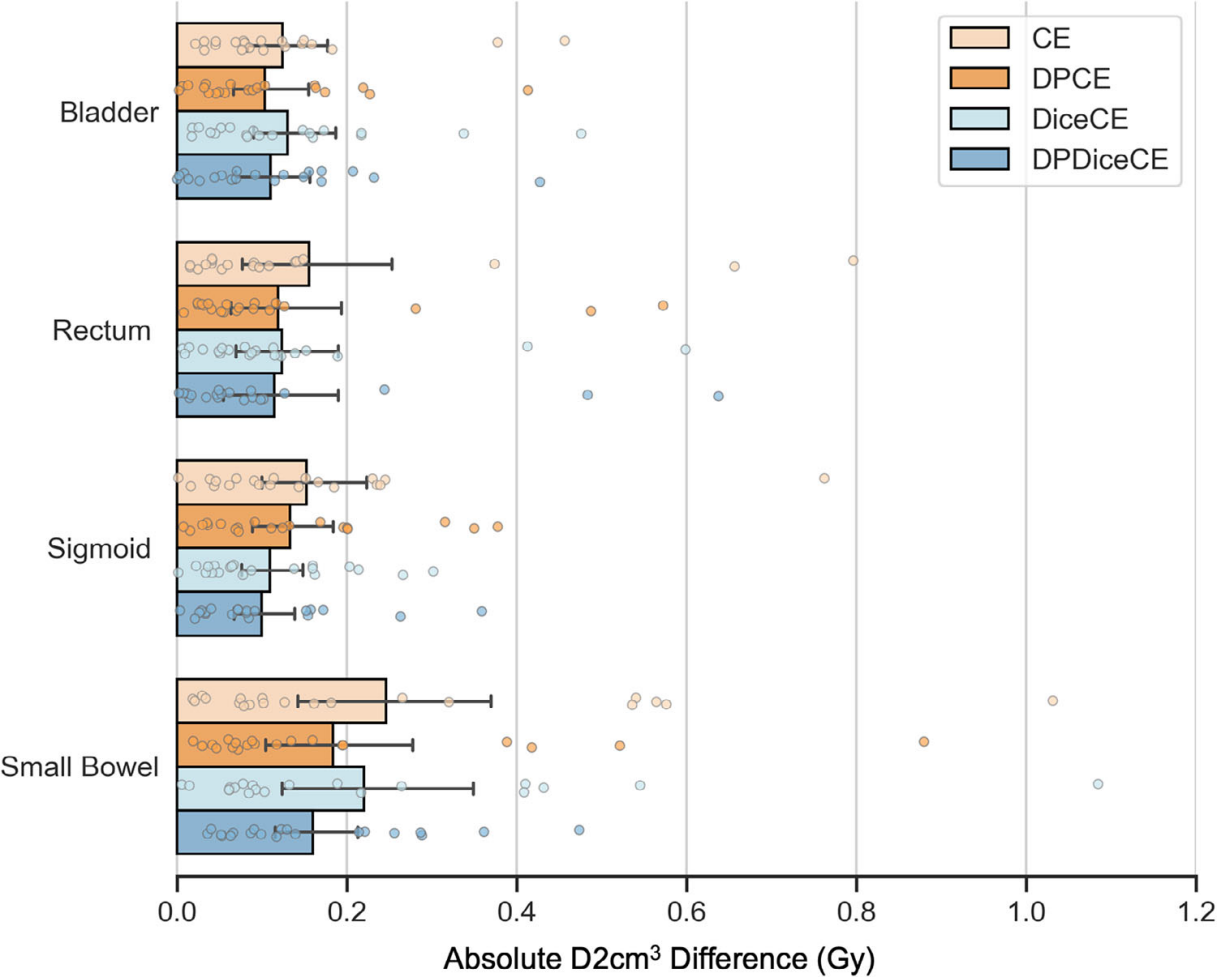
Absolute D2cm^3^ difference in the CE group (orange) and DiceCE group (blue). Absolute D2cm^3^ difference was calculated between the dose of the auto‐contours and the clinical values. Models trained with the distance‐penalized (DP) loss functions (darker bars) had reduced mean absolute D2cm^3^ difference than their non‐DP counterparts (lighter bars). The error bars represent the 95% confidence intervals around the mean.

The models trained with DP loss functions consistently demonstrated reduced mean absolute D2cm^3^ differences than their non‐DP counterparts (Figure 6), indicating enhanced dosimetry accuracy. There was no statistically significant difference between DP loss and its corresponding non‐DP loss with the following *p*‐values: bladder (0.20 and 0.41), rectum (0.19 and 0.60), sigmoid (0.60 and 0.19), and small bowel (0.012 and 0.60) for the CE group and DiceCE group, respectively. The standard deviations of the DPCE loss functions were lower for all four OARs compared to those of the CE loss function, indicating higher consistency in performance. Additionally, the DPDiceCE loss function exhibited smaller standard deviations for the bladder and small bowel when compared to the DiceCE loss function. The D2cm^3^ generated from the auto‐contours aligned with the clinical dose parameters with no statistical difference.

### 3.4 Clinically acceptability test

**FIGURE 7:**
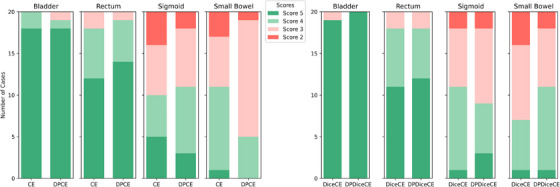
Clinical acceptability ratings between CE and DPCE (left) and between DiceCE and DPDiceCE (right) in four OARs. Clinically acceptable scores (5 or 4) are in green shaded. Not acceptable scores (3 or 2) are in red shaded.

The clinical acceptability ratings (Figure 7) showed that the DP group had marginally superior performance compared to the non‐DP group for some OARs. The model trained with DPCE loss had more clinically acceptable contours (with a score of 5 or 4) than CE loss in two out of four OARs (rectum and sigmoid). There were equal or higher numbers of contours rated as clinically acceptable using the DPDiceCE compared to DiceCE loss, except for the sigmoid.

The average time required for reviewing one scan was 0.73 min (0.2∼3.83 min); the average time to revise the contours in one scan was 4.64 min (0∼19.12 min). For each loss function individually, the mean times for review and revision were similar for non‐DP and DP loss functions. However, a notable difference was observed in the maximum time required for revision. The DP loss functions demonstrated a reduced maximum revision time (12.22 min for DPCE and 10.83 min for DPDiceCE) in comparison to the non‐DP loss functions (19.12 min for CE and 15.27 min for DiceCE).

Physician's revisions had a little impact on D2cm^3^ accuracy (mean D2cm^3^ discrepancy with clinically calculated values before and after revisions <0.05 Gy; Figure S1). Furthermore, our analysis revealed there was no statistically significant linear relationship between the acceptability ratings and the differences in dose parameters before and after revisions (Figure S2) for the bladder, rectum, and small bowel, as evidenced by the results of ordinary least squares (OLS) regression analyses. For sigmoid, while a linear relationship was identified, indicating statistical significance (*p* = 0.006), the model demonstrated minimal explanatory power, with an *R*‐squared value of 0.092.

### 3.5 Independent internal testing

To further evaluate the improvement and generalizability of models trained with the proposed DP loss functions, we performed independent internal testing. After fine‐tuning, models incorporating DP loss functions achieved small but non‐significant improvements in wDSC compared to their non‐DP counterparts (Table S3). Improvements were observed across all OARs, except for the small bowel in the DiceCE group. Similar trends were observed across the five standard evaluation metrics (Table S3).

## 4 DISCUSSION

In this study, we developed and validated a novel evaluation metric and DP loss function in deep learning‐based OAR segmentation for cervical brachytherapy. The newly proposed metric, wDSC, demonstrated the strongest correlation with D2cm^3^ dose difference when compared to five standard geometric measures, thereby suggesting it as the best indicator of D2cm^3^ accuracy. We developed a deep convolutional neural network incorporating the DP loss function to focus the model's attention on near‐to‐target OAR regions, enhancing dosimetric accuracy in brachytherapy. Our model trained with the DP loss function showed improved geometric performance in segmenting OAR regions adjacent to the target, leading to more accurate D2cm^3^ calculations with reduced variations and a decrease in the physician's time required to correct poorly segmented images. Consistent geometric improvement was also shown in the independent internal testing set.

This study is the first study to direct the model's focus toward near‐to‐target OAR regions in radiotherapy. Our results showed that incorporating distance penalties in both CE and DiceCE loss functions improved wDSCs for most organs, suggesting the enhancement of near‐to‐target organ segmentation. Bladder and rectum delineation is generally more straightforward and shows higher inter‐observer agreement among human experts.^25^, ^26^ In contrast, segmenting the sigmoid is more challenging and has higher inter‐observer variability.^25^, ^26^ Previous auto‐segmentation models have also struggled more with the sigmoid as well as the small bowel while achieving relatively accurate results for the bladder and rectum.^27^, ^28^ In our study, we observed that the proposed DP loss function was beneficial for segmenting the more challenging organs—sigmoid and small bowel. We noted greater improvements in median values and overall distributions of the weighted wDSC and $D2cm_{diff}^{3}$. For the small bowel, the improvements were nearly significant after Bonferroni correction. For the bladder and rectum, the DP loss function resulted in only marginal improvements. However, the baseline performance of our model already exceeded reported inter‐observer variability in the literature.^25^ For example, the volume conformity index (VCI) of our model using the CE loss function outperformed values reported by Damato et al. [(mean ± standard deviation): 0.87 ± 0.06 vs. 0.70 ± 0.08 for bladder, and 0.81 ± 0.05 vs. 0.67 ± 0.08 for rectum]. This suggests that the DP loss function is most effective when applied to complex structures with high inter‐observer variability, whereas it has less impact for organs where segmentation is straightforward and reliable. Furthermore, in overall organ segmentation, the models utilizing DP functions demonstrated either comparable or superior performance. This suggests that the integration of distance penalties into the loss function optimizes the segmentation of OAR regions adjacent to the target and preserves the integrity of the entire organ's segmentation accuracy. Compared to the two prior studies,^27^, ^29^ our method achieved comparable or superior whole‐organ segmentation performance.

We further validated our models through dosimetric evaluation and clinical acceptability tests. The dosimetric analyses revealed that the models trained with DP loss functions produced D2cm^3^ that more closely aligned with the clinical dose values. Neither over‐contouring nor under‐contouring was evident when examining the actual $D2cm_{diff}^{3}$ (Figure S3). While some auto‐contours were scored as clinically unacceptable, revisions by the physician resulted in only marginal D2cm^3^ changes, suggesting that the revisions might not alter the dosimetric outcome. Furthermore, we observed no significant correlation between clinical acceptability ratings and the dosimetric changes before and after revisions, implying the criteria for clinical acceptability might not accurately reflect dosimetric precision.

vDSC and HD95 are the frequently utilized metrics for comparing the consistency between the auto‐contours and the ground truth in deep learning studies. Despite their widespread use, these metrics present certain limitations^26^, ^27^, ^28^ and might not adequately capture the clinical objectives, specifically in terms of clinical time saved^11^, ^12^ and dosimetric accuracy^30^ in radiotherapy studies. In EBRT, sDSC and APL have been introduced and validated as metrics offering greater clinical relevance. These metrics are designed to evaluate the overlap between two surfaces, with a particular focus on minor misplacements at OAR borders.^28^, ^29^, ^30^ For evaluating target delineation, McCullum et al. proposed the OAR‐weighted DSC (OAR‐DSC). It was a modified DSC with the consideration of the surrounding OARs and their radiosensitivity.^31^ In the context of brachytherapy, however, the applicability of these metrics might not align with clinical requirements since the contouring accuracy of OAR areas adjacent to the target is of paramount importance for accurate dose calculation. Similarly, in online adaptive radiotherapy, it is crucial to focus on anatomical structures that are likely to affect target regions, ensuring they are accurately segmented.^32^ In the Varian Ethos system, a deep learning‐based auto‐segmentation algorithm is used to delineate these structures (“influencers”) due to their significant impact on target deformation and adaptive plan generation,^14^, ^32^, ^33^ which are typically located in closest proximity to the target.^33^ While these influencers are explicitly contoured, their segmentation accuracy is not evaluated in correlation with their distance to the target.

Taking cervical brachytherapy as an example, our study introduced and validated the wDSC as a new geometric evaluation metric, demonstrating its superior correlation with dosimetry difference compared to existing standard metrics. Our new metric directly correlates the contour geometric accuracy with dosimetry precision, allowing for a fast evaluation of the OAR contour accuracy in the areas pertinent to treatment planning. By being a surrogate for the D2cm^3^ accuracy, wDSC could also help screen for poorly performing cases without the extra step of dosimetric comparison. The pseudo‐dosimetry evaluation may provide a practical approach to assess the model's performance prior to more resource‐intensive analysis. Furthermore, most auto‐segmentation studies in cervical brachytherapy have not included the dosimetric analysis.^34^ We suggest that wDSC may also facilitate comparative evaluation of model performance across different auto‐segmentation studies in a standardized manner. Its application may also be further extended into other brachytherapy sites and online adaptive radiotherapy.

This work has several limitations. First, this was a single institutional study, and the size of our cohort was limited. More data from patients treated in our institution are being collected and will be used to reduce the model bias and further validate our model performance. To preliminarily assess the performance of our DP loss function on different datasets, we conducted independent internal testing using patients treated with a different applicator and scanned with a different scanner. The results were consistent with our original findings, showing small but consistent improvements in near‐to‐target OAR segmentation. In the future study, we intend to validate the model's performance and the impact of wDSC across multiple institutions. Second, wDSC was evaluated exclusively on cervical scans. We aim to expand the applicability of wDSC in brachytherapy by validating it on prostate scans and other high‐dose volumes relevant to prostate brachytherapy. Third, since this study is the first attempt at utilizing the DP loss function and wDSC based on fundamentals of dosimetry using the inverse square law, further modification may improve both training and wDSC agreement to D2cm^3^. Furthermore, our proposed loss function was implemented using only the 3D U‐Net architecture as the network backbone within the nnU‐Net framework. We chose nnU‐Net because it has demonstrated robust and outstanding performance across diverse medical imaging datasets,^35^, ^36^ is self‐configuring, minimizes the need for manual hyperparameter tuning, and is widely used as a baseline model in medical image segmentation studies.^37^, ^38^, ^39^ nnU‐Net automatically adjusts critical architectural parameters of a 3D U‐Net variant, such as patch size, network topology, and training strategies, based on the specific characteristics and spatial dimensions of a given dataset.^21^ While the underlying backbone remains 3D U‐Net, the framework's adaptability to dataset‐specific network properties enables a more comprehensive evaluation of the generalizability of our DP loss function across different datasets. To further assess the robustness of our loss function, we plan to implement it within alternative network architectures, such as transformer‐based models, in future studies. Additionally, our current design for the DP loss function assumes a stable relative distance between the CTV_HR_ and OARs when calculating the DP term in cervical brachytherapy. However, the DP term is adaptable and could be customized for different use cases. In cases where the stable relative assumption does not hold, such as respiratory motion in lung cancer treatment, the reference target selection could be adjusted accordingly. Internal target volume (ITV) could be used instead to account for relative motion between the target and OARs, ensuring accurate distance calculation in motion‐sensitive scenarios. Finally, the clinical acceptability test and contour revisions involved only one radiation oncology fellow. More ROs will be involved to assess the inter‐observer variations and contribute to a more robust evaluation.

## 5 CONCLUSION

This study introduced and evaluated a new geometric metric, wDSC, as a better indicator of D2cm^3^ accuracy in cervical brachytherapy compared to five standard metrics, vDSC, HD95, sDSC, APL, and ASSD. wDSC has the potential to be used as a surrogate of dosimetric accuracy in future auto‐segmentation brachytherapy studies. The model trained with the DP loss function was developed to guide the network's attention toward the near‐to‐target regions. The employment of the DP loss function led to improvements in the mean values of geometric and dosimetric analysis of the auto‐segmented OARs, but these improvements did not reach statistical significance.

## CONFLICT OF INTEREST STATEMENT

Kathy Han received honoraria from Astra Zeneca Cervical Cancer Radiation Oncology Advisory Board and Merck Gynecological Cancers National Consultation Meeting. Jennifer Croke receives payment or honoraria for lectures, presentations, speakers bureaus, manuscript writing or educational events from Merck Canada; she also serves as CARO Annual Scientific Meeting Chair, CARO Equity, Diversity, Inclusion Committee, and ASTRO Equity, Diversity and Inclusion Committee. Benjamin Haibe‐Kains is a consultant and shareholder of Code Ocean Inc.

## Supporting information



Supporting Information
