# Supplementary material for: Geometrically focused training and evaluation of organs‐at‐risk segmentation via deep learning
Source: Med Phys. 2025 Apr 25;52(7):e17840. doi: 10.1002/mp.17840 (PMC12257911; doi:10.1002/mp.17840)
Supplement: Supplementary file 1 — Supporting Information [file MP-52-0-s001.docx]

**Supplementary Table 1**. Five-point rating scale and description for the clinical acceptability test^22^. A contour with a score of 5 or 4 is clinically acceptable.

| **Score** | | **Acceptability** | **Description** |
| --- | --- | --- | --- |
| 5 | Acceptable, Use-as-is | | Clinically acceptable, could be used for planning without changes |
| 4 | Acceptable, minor edits but not necessary | | Within acceptable inter-observer variation for planning |
| 3 | Unacceptable, minor edits are necessary | | Minor edits are required, but it is more efficient to edit the auto-generated contours than start from scratch |
| 2 | Unacceptable, major edits are necessary | | Major edits are required for planning purpose and user would prefer to start from scratch |
| 1 | Unacceptable, unusable | | Auto-generated contours are unusable for planning purpose |

**Supplementary Table 2**. Standard metrics comparing the performance of four loss functions across all OARs. All values are presented as mean ± standard deviation. Bold indicates the best performance of one metric among the four loss functions.

|  |  | Bladder | Rectum | Sigmoid | Small Bowel |
| --- | --- | --- | --- | --- | --- |
| vDSC ↑ | CE | 0.93 ± 0.03 | 0.89 ± 0.03 | 0.7 ± 0.2 | 0.7 ± 0.1 |
|  | DPCE | **0.94** ± 0.03 | **0.90** ± 0.03 | 0.7 ± 0.2 | 0.7 ± 0.1 |
|  | DiceCE | **0.94** ± 0.03 | 0.89 ± 0.03 | 0.8 ± 0.2 | 0.7 ± 0.1 |
|  | DPDiceCE | 0.93 ± 0.03 | **0.90** ± 0.03 | **0.8** ± 0.2 | **0.8** ± 0.1 |
| HD95 (mm) ↓ | CE | 3 ± 2 | 5 ± 3 | 20 ± 20 | 25 ± 16 |
|  | DPCE | **2.8** ± 0.9 | **5** ± 3 | 21 ± 17 | 23 ± 17 |
|  | DiceCE | 4 ± 8 | 5 ± 2 | 19 ± 16 | 24 ± 17 |
|  | DPDiceCE | 3 ± 1 | 5 ± 2 | **17** ± 16 | **19** ± 15 |
| sDSC ↑ | CE | 0.78 ± 0.07 | 0.79 ± 0.07 | 0.6 ± 0.2 | 0.50 ± 0.08 |
|  | DPCE | **0.82** ± 0.06 | **0.81** ± 0.08 | 0.7 ± 0.2 | 0.52 ± 0.09 |
|  | DiceCE | 0.81 ± 0.06 | 0.80 ± 0.09 | 0.7 ± 0.1 | 0.52 ± 0.08 |
|  | DPDiceCE | 0.81 ± 0.06 | 0.80 ± 0.08 | **0.7** ± 0.1 | **0.55** ± 0.09 |
| APL (cm) ↓ | CE | 12 ± 4 | 8 ± 3 | 19 ± 12 | 30 ± 20 |
|  | DPCE | **11** ± 3 | **8** ± 3 | 19 ± 12 | 30 ± 20 |
|  | DiceCE | 12 ± 4 | 8 ± 3 | 18 ± 12 | 30 ± 20 |
|  | DPDiceCE | 12 ± 3 | 8 ± 3 | **18** ± 11 | 30 ± 20 |
| ASSD (mm) ↓ | CE | 0.7 ± 0.3 | 0.8 ± 0.3 | 3 ± 4 | 4 ± 2 |
|  | DPCE | **0.6 ±** 0.2 | **0.8** ± 0.3 | 4 ± 4 | 4 ± 2 |
|  | DiceCE | 0.7 ± 0.3 | 0.9 ± 0.5 | 3 ± 4 | 4 ± 2 |
|  | DPDiceCE | 0.6 **±** 0.2 | 0.8 ± 0.4 | **3** ± 4 | **3** ± 2 |


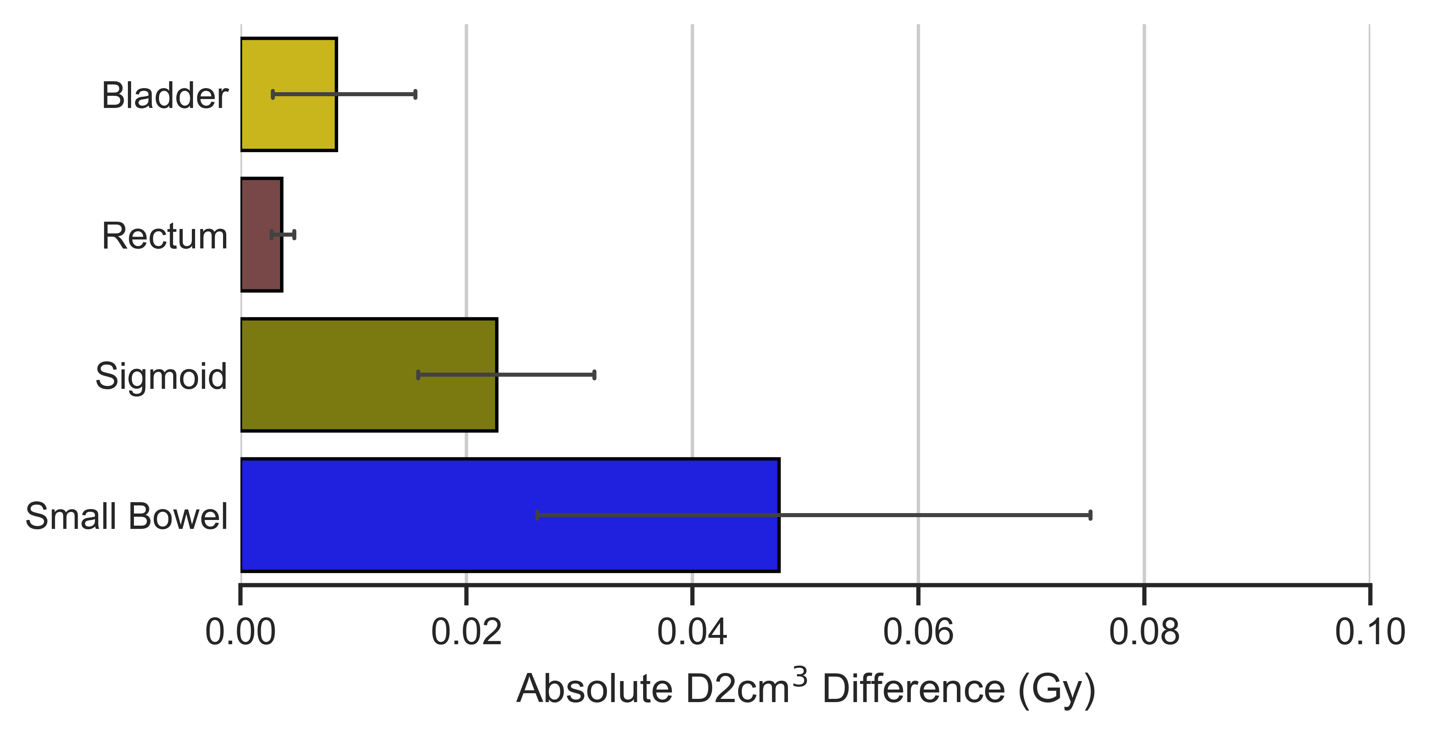
**Supplementary Figure 1**. Absolute D2cm^3^ difference before and after physician's revisions for each OAR. Error bars represent the standard deviations.


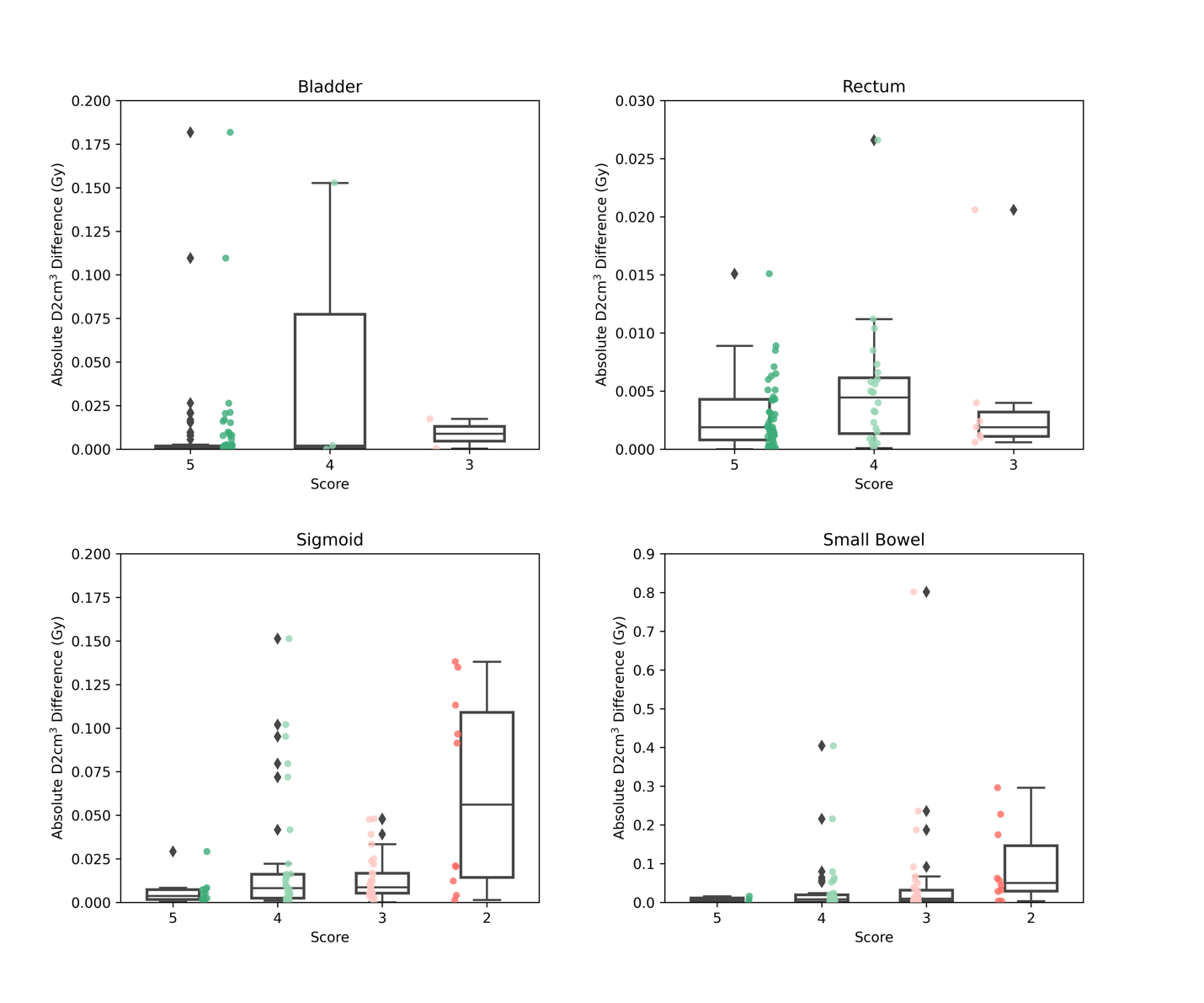
**Supplementary Figure 2**. Correlations between absolute D2cm^3^ difference and the clinical acceptability ratings for four OARs. The absolute D2cm^3^ difference was calculated between the D2cm^3^ before and after the physician's revisions to show the impact of the revisions.

**Supplementary Table 3**. The comparisons of wDSC and standard metrics of four loss functions in independent internal testing dataset (10 scans). All values are presented as mean ± standard deviation. Bold indicates the best performance of one metric among the four loss functions.

|  |  | Bladder | Rectum | Sigmoid | Small Bowel |
| --- | --- | --- | --- | --- | --- |
| wDSC ↑ | CE | 0.95 ± 0.03 | 0.91 ± 0.04 | 0.84 ± 0.09 | 0.8 ± 0.1 |
|  | DPCE | 0.95 ± 0.03 | 0.92 ± 0.03 | 0.85 ± 0.09 | 0.80 ± 0.08 |
|  | DiceCE | 0.95 ± 0.04 | 0.92 ± 0.04 | 0.85 ± 0.09 | **0.82** ± 0.09 |
|  | DPDiceCE | **0.95** ± 0.02 | **0.93** ± 0.03 | **0.86** ± 0.06 | 0.81 ± 0.08 |
| vDSC ↑ | CE | 0.94 ± 0.04 | 0.85 ± 0.07 | 0.7 ± 0.1 | 0.7 ± 0.3 |
|  | DPCE | 0.93 ± 0.05 | 0.86 ± 0.05 | 0.7 ± 0.1 | 0.7 ± 0.2 |
|  | DiceCE | 0.93 ± 0.06 | 0.86 ± 0.06 | 0.7 ± 0.2 | **0.7** ± 0.3 |
|  | DPDiceCE | **0.94** ± 0.04 | **0.87** ± 0.06 | **0.7** ± 0.1 | 0.7 ± 0.2 |
| HD95 (mm) ↓ | CE | 20 ± 40 | 20 ± 30 | 30 ± 40 | 30 ± 50 |
|  | DPCE | 20 ± 40 | 10 ± 7 | 30 ± 30 | **20** ± 20 |
|  | DiceCE | 20 ± 30 | 9 ± 8 | 30 ± 40 | 30 ± 60 |
|  | DPDiceCE | **7** ± 13 | **8** ± 7 | **20** ± 18 | 20 ± 20 |
| sDSC ↑ | CE | 0.8 ± 0.1 | 0.7 ± 0.1 | 0.6 ± 0.2 | 0.5 ± 0.2 |
|  | DPCE | 0.8 ± 0.1 | 0.7 ± 0.1 | 0.6 ± 0.2 | 0.5 ± 0.2 |
|  | DiceCE | 0.8 ± 0.1 | 0.7 ± 0.1 | **0.6** ± 0.2 | 0.5 ± 0.2 |
|  | DPDiceCE | **0.8** ± 0.1 | **0.8** ± 0.1 | 0.6 ± 0.2 | **0.5** ± 0.2 |
| APL (cm) ↓ | CE | 14 ± 3 | 9 ± 3 | 16 ± 11 | 40 ± 19 |
|  | DPCE | 14 ± 3 | **9** ± 3 | 17 ± 12 | 40 ± 18 |
|  | DiceCE | **14** ± 3 | 9 ± 3 | **16** ± 13 | 40 ± 19 |
|  | DPDiceCE | 14 ± 3 | 9 ± 3 | 17 ± 12 | **30** ± 17 |
| ASSD (mm) ↓ | CE | 2 ± 4 | 2 ± 1 | 4 ± 4 | 5 ± 9 |
|  | DPCE | 2 ± 3 | 1.5 ± 0.9 | 4 ± 2 | **4** ± 5 |
|  | DiceCE | 3 ± 5 | 1 ± 1 | 4 ± 5 | 6 ± 11 |
|  | DPDiceCE | **0.9** ± 0.9 | **1** ± 1 | **3** ± 2 | 4 ± 5 |

**
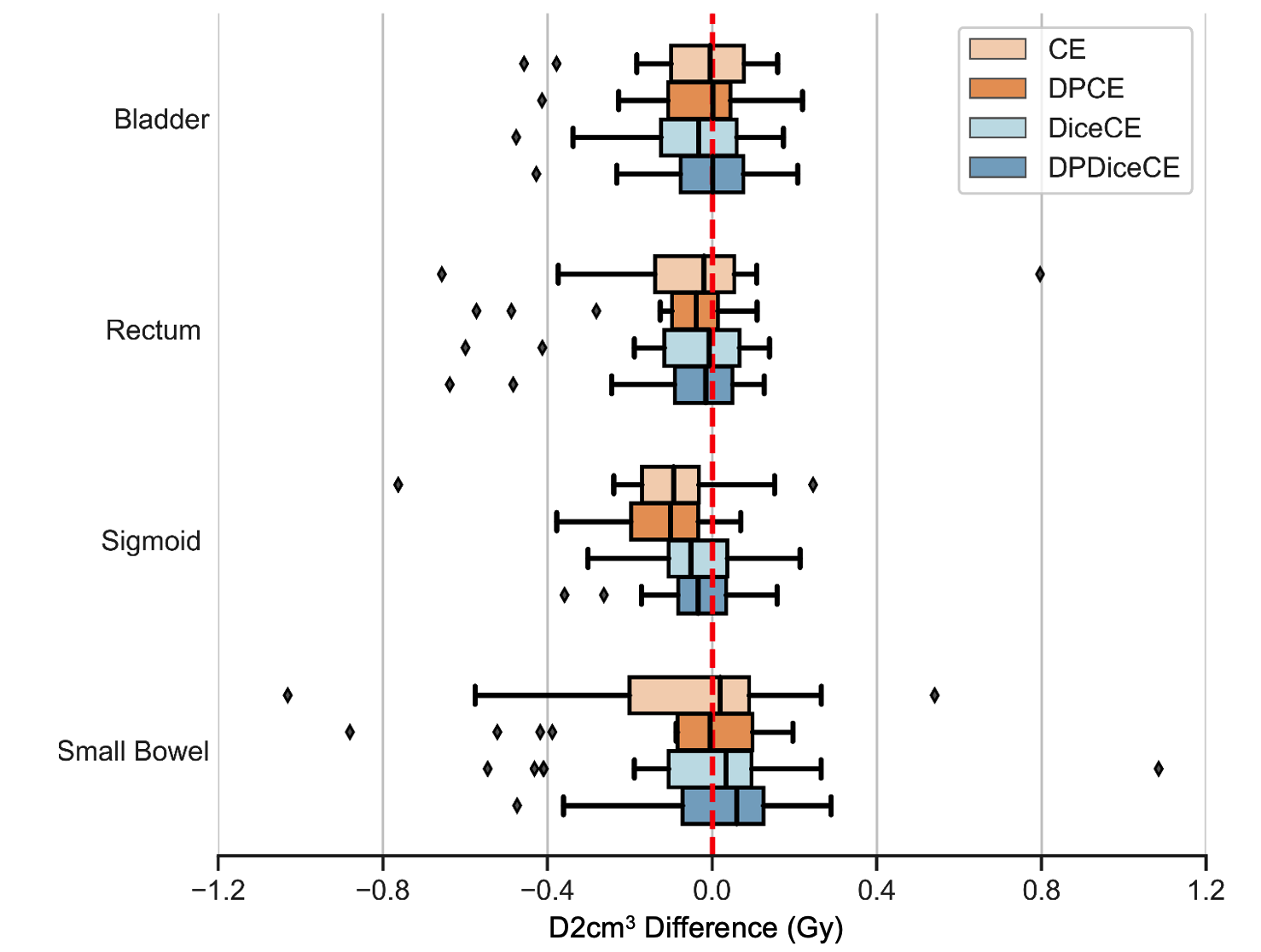
Supplementary Figure 3**. D2cm^3^ difference (actual values; not absolute) in the CE group (orange) and DiceCE group (blue). CE: cross-entropy loss; DPCE: distance-penalized cross-entropy loss; DiceCE: Dice+cross-entropy loss; DPDiceCE: distance-penalized dice+cross-entropy loss.


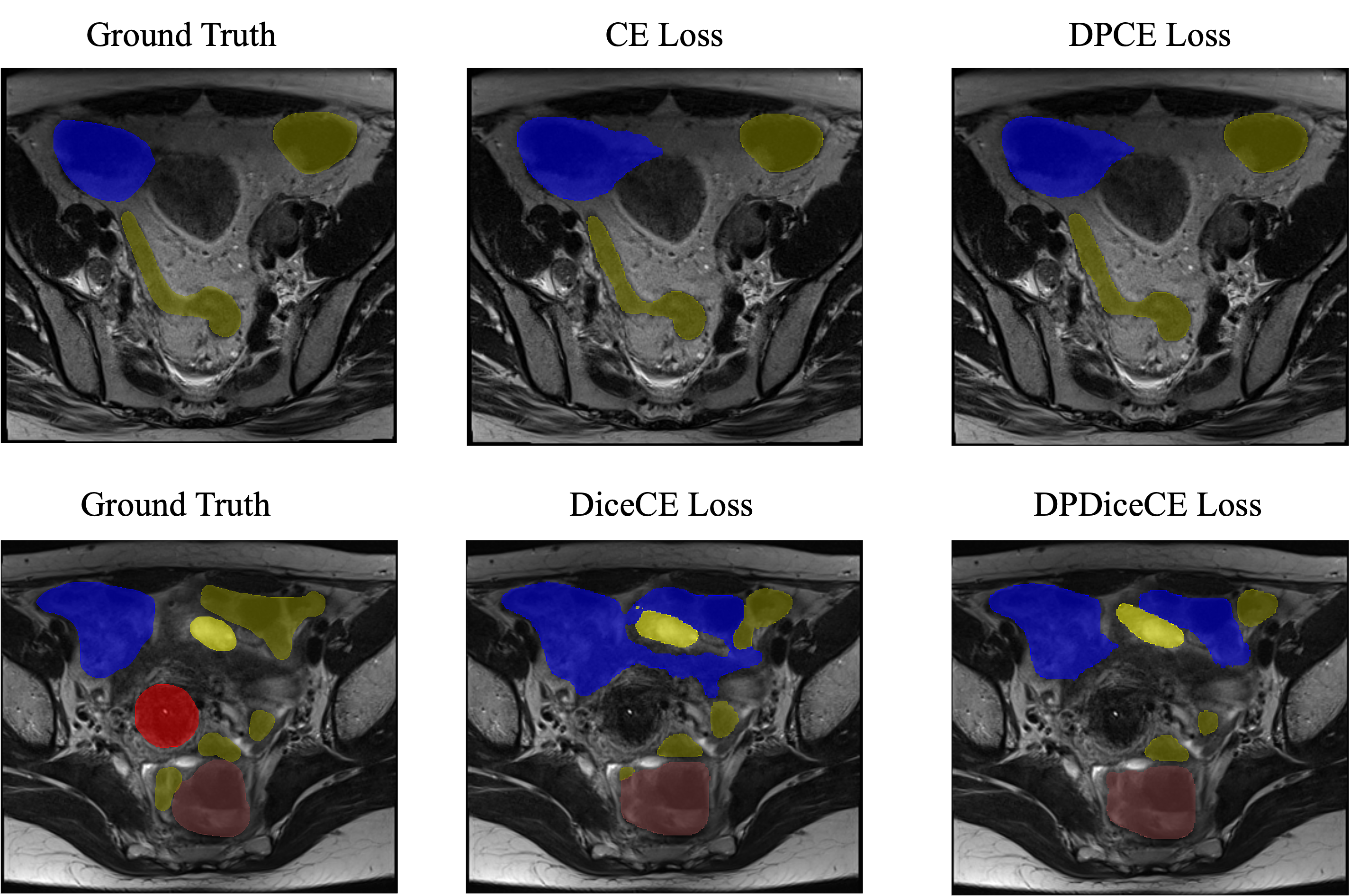


**Supplementary Figure 4**. Examples of axial contours from CE loss, DPCE loss, DiceCE loss, and DPDiceCE loss. Bladder (yellow), rectum (brown), sigmoid (dark yellow), and small bowel (blue) are depicted. The HR-CTV ground truth (red) is shown to demonstrate the target location.
